# What is the result of vaginal cleansing with chlorhexidine during labour on maternal and neonatal infections? A systematic review of randomised trials with meta-analysis

**DOI:** 10.1186/s12884-018-1754-9

**Published:** 2018-05-08

**Authors:** Charlotte Bell, Laura Hughes, Trevor Akister, Vin Ramkhelawon, Amie Wilson, David Lissauer

**Affiliations:** 1 South Warwickshire NHS Foundation Trust, Lakin Road, Warwick, CV34 5BW UK; 2 Wye Valley NHS Trust, The County Hospital, Hereford, HR1 2BN UK; 3 Sandwell and West Birmingham Hospitals NHS Trust, Dudley Road, Birmingham, B18 7QH UK; 4 Institute of Applied Health Research, University of Birmingham, B15 2TT, Birmingham, UK; 5 Institute of Metabolism and Systems Research, College of Medical and Dental Sciences, University of Birmingham, B15 2TT, Edgbaston, UK

**Keywords:** Maternal, Chlorhexidine, Infection, Systematic review, Neonatal, Infection prevention

## Abstract

**Background:**

Infection with vaginal microorganisms during labour can lead to maternal and neonatal mortality and morbidity.

The objective of this systematic review is to review the effectiveness of intrapartum vaginal chlorhexidine in the reduction of maternal and neonatal colonisation and infectious morbidity.

**Methods:**

**Search strategy –** Eight databases were searched for articles published in any language from inception to October 2016.

**Selection criteria –** Randomised controlled trials were included.

**Data Collection and analysis** - Publications were assessed for inclusion. Data were extracted and assessed for risk of bias.

Relative risks from individual studies were pooled using a random effects model and the heterogeneity of treatment was evaluated using Chi^2^ and *I*^2^ tests.

**Results:**

Eleven randomised controlled trials (*n* = 20,101) evaluated intrapartum vaginal chlorhexidine interventions. Meta-analysis found no significant differences between the intervention and control groups for any of the four outcomes: maternal or neonatal colonization or infection. The preferred method for chlorhexidine administration was vaginal irrigation.

**Conclusions:**

Meta-analysis did not demonstrate improved maternal or neonatal outcomes with intrapartum vaginal chlorhexidine cleansing, however this may be due to the limitations of the available studies. A larger, multicentre randomised controlled trial, powered to accurately evaluate the effect of intrapartum vaginal chlorhexidine cleansing on neonatal outcomes may still be informative; the technique of douching may be the most promising.

## Background

Maternal and neonatal morbidity and mortality continue to present a serious global problem. In 2015 over 137 million live births were estimated worldwide [1], and 2.7 million neonatal deaths. [1]., A further 303,000 maternal deaths were recorded in 2015 [2].

Between 30 and 40% of neonatal deaths worldwide are caused by infections [3, 4] and 10.7% of maternal deaths (37,285 annually worldwide) are due to sepsis [5]. The greatest burden exists in low-income countries, where 99% of neonatal and maternal deaths occur [6, 7]. Therefore, in order for interventions to have real potential for benefit, it is imperative that they are easily accessible, both financially and in practical application.

During the process of labour, both mother and fetus are susceptible to infection from a range of vaginal microorganisms including Group B streptococci (GBS), Campylobacter, *Enterococcus faecalis*, *methicillin-resistant Streptococcus aureus*, *Klebsiellapneumoniae*, *Escherichia coli* and *Acinetobaumannii* [8]. These organisms can lead to maternal and neonatal mortality and morbidities such as septicaemia, meningitis and pneumonia in the neonate [9] and chorioamnionitis leading to severe pelvic infection in the mother [10].

The maternal and fetal microbial profile may differ between geographical regions, with GBS having prominence in high-income countries [11]. However, it has been hypothesised that this prominence may be due to the underestimation of GBS prevalence in low income countries; facilities for detection are rarely available and many births take place outside a formal healthcare setting [12]. Thus far, many studies have focused separately on GBS and other vaginal microbes [9, 13–22].

GBS in the neonate is usually acquired through vertical transmission from the mother’s genital tract [23]. A number of strategies have been suggested to reduce vertical transmission of pathogens which colonise the maternal genital tract [13], including the use of intrapartum chemoprophylaxis for GBS-colonised mothers [24] and whole-body washing with chlorhexidine during the last 2 weeks of pregnancy [14]. In particular an important research question has been the use of a chlorhexidine antiseptic to cleanse the vagina during labour to reduce both maternal and neonatal infection [15, 20, 25–30].

Chlorhexidine is a bisguanide antiseptic, which works by disrupting the bacterial cell wall [31]. It is effective against most gram-positive and some gram-negative bacteria, yeasts and many viruses, although variably effective against enveloped viruses [31]. It is ineffective against bacterial spores and mycobacteria [31]. Christensen et al. [13] found that GBS was extremely sensitive to chlorhexidine, with a minimum inhibitory concentration of 0.5-1 mg/l [32].Chlorhexidine has been shown to have activity against normal vaginal bacteria, which cause puerperal infection, including GBS, *E.coli* and enterococci [33]. Upon application it is immediately effective, suppressing bacterial growth for up to 24 h [15]. Although not deactivated by alcohol, soaps or lavage fluid, the presence of organic matter such as blood or amniotic fluid may reduce the effectiveness of chlorhexidine [31].

The broad-spectrum antisepsis of the compound makes it particularly suitable for use in the intrapartum environment, where the colonisation of neonates and infectious morbidity of mothers shows an ever-changing pattern [34]. It is effective at a lower pH, which further supports its use in the vagina, which typically has an environment of pH < 4.7 [35].Chlorhexidine is inexpensive, has no effect on antimicrobial resistance, and is practical and viable to be used in resource-limited settings [36]. It also has a good safety profile [37] and has been studied in the obstetric setting in concentrations ranging from 0.05–4% [11] The compound is widely available from numerous manufacturers worldwide. Chlorhexidine has thus been proposed as a highly suitable compound for intra-vaginal use to reduce maternal and neonatal sepsis [12, 38].

In 1989, the observation of a reduction of neonatal GBS colonisation led to the recommendation for a larger multicentre trial [16]. More recently, two Cochrane reviews of randomised controlled trials examined aspects of this question [17, 18] both of which were updated in 2014 [9, 19]. Lumbiganon et al. [9] reported data in their Cochrane review which focused on trials comparing chlorhexidine vaginal douching during labour with placebo or other vaginal disinfectant to prevent maternal and neonatal infections, excluding GBS and HIV. The results suggested a trend in the reduction of endometritis through intrapartum vaginal chlorhexidine, but this was not statistically significant. Ohlsson et al. [19] found that a vaginal intrapartum chlorhexidine intervention reduced the GBS colonisation of neonates, but did not reduce early-onset disease, including GBS infection, GBS pneumonia or GBS meningitis. The authors of both reviews concluded that a randomised controlled trial with adequate power and standardised intervention was required, but Ohlsson et al. [19] commented that in developed countries, this may be difficult to justify in the era of antibiotic prophylaxis for GBS infection. However, the scope of these reviews was narrower than this review, and excluded a number studies as they combined the interventions of vaginal cleansing and infant washing. Furthermore the Cochrane reviews separated neonatal infections based on the microorganism responsible, making an overall assessment of the efficacy of this intervention difficult.

The following systematic review and meta-analysis of randomised controlled trials focuses on the intrapartum vaginal interventions in vaginal deliveries only, measuring both maternal and neonatal outcomes in terms of infectious morbidity and mortality, irrespective of infectious organisms.

## Methods

Types of studies included randomised controlled trials only, comparing the use of intrapartum vaginal chlorhexidine cleansing to no chlorhexidine use or placebo or other vaginal disinfectant, for the reduction of maternal or neonatal infection. Studies that considered HIV-positive participants exclusively were excluded.

Participants considered for inclusion in this review are women undergoing vaginal delivery, in the intrapartum period and having vaginal chlorhexidine cleansing in any setting.

Types of interventions considered were vaginal disinfection with chlorhexidine by any method during labour, compared with placebo or no vaginal disinfection.

Maternal outcomes measured were 1) Colonization during the post-partum period and 2) Clinical infection and / or sepsis during the post-partum period. Neonatal outcomes measured were 1) Colonization during the neonatal period and 2) Clinical infection and / or sepsis during the neonatal period.

Eight electronic databases were searched (PubMed, Medline, Embase, Cochrane Central Register of Controlled Trials, CINAHL, AIM, the Reproductive Health Library, and BioMed Central: from database inception to 10/2016. The following search terms were used ‘Chlorhexidine’, ‘vaginal antiseptic’, ‘vaginal wipe’, ‘vaginal douche’, ‘vaginal cleansing’, ‘bathing’ with ‘pregnancy’, ‘postpartum’, ‘labour’ ‘intrapartum’, ‘neonatal’, ‘peripartum’ and ‘meningitis’, ‘pneumonia’ ‘group B strep’, ‘infection’, ‘HIV’, ‘sepsis’, ‘mortality’, ‘omphalitis’, ‘Klebsiella’, ‘chorioamnionitis’, ‘endometritis’, ‘maternal’, ‘infant’, ‘postnatal’. No language restrictions were applied. Databases were searched for papers published until October 2016.

All randomised trials examining the use of vaginal chlorhexidine washing during labour, by any method, which reported maternal or neonatal outcomes were included.

Three authors completed the searches independently (C Bell, L Hughes, T Akister). Two authors independently (C Bell, L Hughes) screened the titles and abstracts to assess for inclusion or exclusion. The two authors then read each paper identified as a result of the search strategy and made a decision on whether it should be included or excluded on the basis of all the defined inclusion criteria. Disagreements were resolved by discussion (T Akister, D Lissauer).

Data was extracted by two authors independently (T Akister, V Ramkhelawon) and tabulated using Miscrosoft Excel. Any disagreements were resolved by discussion amongst the authorship group and consensus. Data was entered into Review Manager Software Revman 5.0 and checked for accuracy.

Two review authors (T Akister, V Ramkhelawon) independently assessed risk of bias for each study using the criteria outlined in the *Cochrane Handbook for Systematic Reviews of Interventions* [39]. Any disagreement was resolved by discussion or by involving a third review author.

Specifically, the following aspects of risk bias were assessed in detail: 1) Sequence generation (checking for possible selection bias), 2) Allocation concealment (checking for possible selection bias), 3) Blinding (checking for possible performance bias), 4) Incomplete outcome data (checking for possible attrition bias through withdrawals, dropouts, protocol deviations), 5) Selective reporting bias, 6) Other sources of bias.

The overall risk of bias was made using judgements about whether studies were at high risk of bias, according to the criteria given in the *Cochrane Handbook for Systematic Reviews of Interventions* [39]. The likely magnitude and direction of the biases described in points 1 to 6 above was assessed and whether it was likely to impact on the findings.

Data for effect estimates, including 95% confidence intervals, were directly extracted. These results were then included in the meta-analysis, using a random effects model to pool the relative risks from individual studies. The heterogeneity of treatment was evaluated using Chi^2^ and *I*^2^ tests and presented as forest plots. Analyses were undertaken using Revman 5.0 statistical software and Mantel-Haenszel analysis.

## Results

**Fig. 1:**
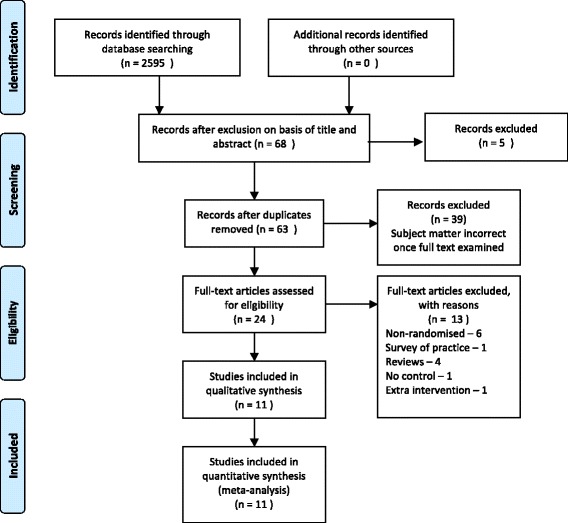
PRISMA flow diagram of search strategy and process of selecting articles

We identified 68 unique papers after searching PubMed, Embase, Medline, The Cochrane Library and Biomed Central. No papers were identified after searching the CINAHL, AIM or RHL databases. Eleven RCTs involving 20,101 women and their infants, were suitable to be included in a systematic review and meta-analysis (Fig. 1). Characteristics of included studies are detailed in Table 1, including potential confounding factors. Only two of the studies [27, 40] were undertaken in low resource settings (Table 1).

**Table 1:** Characteristics of studies included in meta-analysis

Study, Country	Details	Population	Criteria for exclusion from study	Characteristics of mothers	Characteristics of neonates	Potential confounders	Intervention	Control	Number of participants	Outcomes
Adriaanse et al 1995, Holland	At onset of labour, the attending obstetrician applied 10 ml chlorhexidine (CHX) gel around the portio vaginalis and into the fornices. This procedure was repeated after 10 h in case delivery had not yet occurred.	Pregnant women from two hospitals with obstetric services in the city of Nijmegen, the Netherlands.	Known GBS carrier, use of antibiotics during the 4 weeks before admission, planned caesarean section, antepartum foetal death, suspected congenital abnormalities and premature labour.	No significant difference between groups	No significant differences between the three groups, except for the % of neonates admitted to the (special) neonatal care unit (P = 0.012) in the CHX and control group.	No special training given to doctors giving intervention, no protocol given for intervention e.g. timing of washing.	10ml 0.3% CHX gel	Standard care	1020 participating women, 522 were enrolled in one hospital and 498 in the other. Of the 981 analysed mother-infant pairs, 327 were assigned to the chlorhexidine group, 328 to the placebo group and 326 to the control group.	Primary outcomes were vertical GBS transmission to the neonate. Secondary goals were to study the vertical transmission rates of *E. coli, S. aureus* and *C. albicans*, and to establish neonatal and maternal morbidity. Neonatal septicaemia, meningitis and pneumonia diagnosed from the positive cultures of blood or CSF or tracheal aspirate.
Burman 1992, Sweden	60mls of solution (CHX or sterile water) was used to flush the anterior fornix, vaginal walls and urethral orifice in a spiral outward motion by a midwife. This was repeated every 6hours until delivery. Flush was counted if birth occurred > 1 hour after flush and no more than 6 hours lapsed between flushes.	Pregnant women who were urogenital carriers of GBS from 10 Swedish hospitals.	Pre term infants (<37 weeks) planned caesarean section, pregnancy complications after the 30th week of gestation requiring hospital admission, twin or multiple pregnancies, suspected congenital abnormality of the infant, known or suspected allergy to CHX, previous invasive GBS infection, antibiotics during the 2 weeks before admission, and antepartum foetal death.	Not analysed	No significant differences seen	No rigorous set procedure for flushing e.g. time taken to flush, no specialist training given to midwives, multiparity pregnancies excluded, group sizes not even, maternal characteristics not determined.	60 ml 2g/l CHX given as 2 30ml ampules via catheter	60ml sterile saline	4483 women 2238 CHX group and2245 saline placebo group	Rate of admission of babies to special-care neonatal units within 48 h of delivery. Admissions for sepsis/meningitis, pneumonia, skin infection, meconium aspiration, surveillance, maladaptation and non-specific problems were included.
Cutland et al 2009 South Africa	Midwives wrapped cotton pads soaked in water or CHX around gloved fingers. Fingers were rotated circumferentially over the cervix and vaginal walls, and the external genitalia wiped	Pregnant women (Aged 15-51) and their neonates born to South African women at Chris Hani- Baragwanathe hospital, Soweto, South Africa	Exclusion criteria were planned caesarean section, antepartum haemorrhage, known severe congenital malformation, intrauterine death, allergy to CHX, face presentation, genital warts or ulcers, full cervical dilatation, and age younger than 15 years.	No significant differences seen	No significant differences seen	No volumes or times of washing stated	0.5% CHX	Autoclaved tap water	8011 mothers 4005 to chx and 4006 to control	Neonatal and maternal Sepsis and vertical transmission GBS within 1st 3 days of life. Neonatal sepsis defined as clinical diagnosis or culture positive. Maternal sepsis defined as admission within 14 days of delivery for endometritis (at least two of uterine tenderness, fever, foul-smelling or purulent lochia, or vaginal discharge), culture confirmed infection of sterile site, or perineal wound infection among vaginal parturients.
Dykes et al 1987, Sweden	Midwives used a compress steeped in the 2g/L CHX solution. Compress turned three times around the cervix then over the vaginal walls using spiral movements outwards. Procedure was repeated twice with new compresses. Fourth compress was pressed against the cervical orifice and then used for washing of labia minora and the introitus. All patients including those in the control group also had a shower using soap containing CHX on admission and had their lower abdomen and external genitalia washed with CHX prior to delivery.	All pregnant women attending the antenatal clinics in the region served by the Department of Obstetrics, University Hospital, Lund, who were GBS positive (urogenital tract) at weeks 32 and 36 and at onset of labour.	Not stated	Not analysed	Not analysed	All participants had CHX wash including controls, no exclusion criteria e.g. for prior antibiotic use.	2g/l CHX	Standard care	78 patients in total 31 in chx and 47 in the control group.	Maternal urogenital colonisation GBS at 4 days post-partum.
Eriksen et al 1997, USA	20 cc of a 0.4% CHX solution was placed around the portio vaginalis and fornices using a syringe. Women in the control group were irrigated with 20 cc of sterile water.	Women admitted to the Lyndon Baines Johnson Hospital, Texas, USA labour and delivery room	Preterm labour, foetal distress, malpresentation, intraamniotic infection, cervical dilatation >6 cm, and known allergy to CHX.	Not reported	Not reported	Patients with prior use of antibiotics not excluded, no protocol for washing procedure	20cc 0.4% CHX	20cc sterile water	947 patients were randomized to CHX (481) or of sterile water (466)	Incidence of neonatal pneumonia, culture proven neonatal sepsis, and use of the antibiotics in the neonate. The diagnosis of neonatal pneumonia was made by the attending physician if the neonate was febrile and had chest radiograph findings consistent with the diagnosis. Neonatal sepsis was diagnosed if the infant had a positive blood or CSF culture, along with a clinical course consistent with sepsis.
Hennequin 1995, Denmark	Vaginal examinations of the treated group were systematically per- formed with gloves lubrified with 5 ml CHX digluconate 1% cream; the control group was examined with uncoated gloves.	Pregnant antenatally screened GBS positive pregnant women attending the labour ward	Not stated	Not reported	Not reported	No exclusion criteria e.g. abx use ruptured membranes etc, no protocol for vaginal examination, no training given	5 ml CHX digluconate 1% cream	Standard care	59 women in total. 28 CHX cream 31 control	Mother Infant GBS transmission.
Pereira et al 2011, Zimbabwe	Vulva cleansing with a 4x4 cotton wool ball soaked in 15-20ml 1% CHX solution followed by vaginal cleansing with another cotton wool ball as described above. The process was repeated from onset every 2 hours.	Pregnant women attending Harare central hospital who had no allergy to CHX, lived in close proximity to the hospital and planned to have a vaginal birth.	None stated	No significant difference between groups	Apgar scores were significantly higher in CHX group. However neonatal outcomes not included as had full body washing.	No exclusion criteria, no training given,	15-20ml 1% CHX	Standard care	502 women in total 2:1 randomisation 334 to chx and 168 to UC. However only 37 women were swabbed for cultures. 5 in UC 32 in chx.	Safety, acceptability and antimicrobial effect of 1% CHX. Maternal vaginal colonisation (any species) was primary antimicrobial effect measured
Rouse et al 1997, USA	Irrigations were performed either during active labour or before planned caesarean delivery by resident physicians and medical students. CHX solution containing bottles were aseptically attached to 12 cm douche nozzles. These were inserted high into the vaginal fornix, and, as completely as possible, discharged the contents of the bottles. Typically, approximately 200 ml of the irrigation was delivered.	Pregnant women at 24 weeks gestation or more at Cooper Green Hospital, hospital in Birmingham, Alabama, serving publicly funded patients	Contraindication to digital cervical examination (e.g., placenta previa), active genital herpes, chorioamnionitis before randomization, or known or suspected allergy to CHX	Significant differences seen in maternal age, nulliparous, meconium and Intrauterine pressure catheter	Not analysed	Prophylactic antibiotics given for early onset neonatal group B streptococcal sepsis for the following risk factors: anticipated delivery before 37 weeks, rupture of membranes >18 hours, history of a prior affected neonate, or intrapartum fever (temperature - >100.0 ° F)	0.2% CHX	200ml sterile water vaginal wash out pre delivery	A total of 1024 patients were enrolled: 508 in the CHX group and 516 in the placebo group.	Primary outcomes Maternal chorioamnionitis and endometritis Other outcomes; UTI and wound infection Neonatal outcomes; Sepsis, hyperbilirubinaemia, Death, necrotizing enterocolitis, supplemental oxygen, APGAR and intraventricular haemorrhage.
Rouse et al 2003, USA	See Rouse 1997 performed every 6 hours (maximum 4 irrigations)	See Rouse 1997. Patients were eligible if they were nulliparous and admitted for delivery at 32 weeks of gestation	See Rouse 1997	No significant difference between groups seen.	Not analysed	Prophylactic antibiotics given See Rouse 1997	See Rouse 1997	See Rouse 1997	1041 participants 525 in chx; 516 in control	Primary outcomes: Maternal infection - chorioamnionitis and endometritis Secondary neonatal outcomes included birth weight, Apgar scores <7, receipt of antibiotics, need for mechanical ventilation, and admission to the neonatal intensive care unit
Stray- Pedersen et al 1999, Norway	Douching started by intravaginal insertion of catheter towards the cervix. The bottle was squeezed while the catheter was retracted slowly. Patient remained supine for 5 min. Process repeated every 6 hours.	Over 9 Months pregnant women were consecutively selected from the Aker University Hospital, Norway. The first 4 months was a reference period and the next five months the intervention period.	None given	No significant difference between groups	No significant difference between groups	Ampicillin was given to women with prolonged delivery > 24 hours	120 ml 0.2% CHX douche	Reference phase standard care. Intervention phase vaginal douche with sterile saline	1989 participants 548 in chx douche 583 control (saline douche) 858 reference group (nothing)	GBS transmission, Maternal outcomes (postpartum UTI and fever) Fever was recorded when temperature exceeded 38.5°C during the first 24 h after delivery, or if the temperature thereafter exceeded 38°C on two occasions at least 4 h apart, provided that other obvious explanations were absent. Neonatal outcomes (Septicaemia, Strep. agalactiae sepsis Respiratory problems and Superficial infections)
Sweeten et al 1997, USA	Women randomized to the study arm received 20 ml of a 0.4% CHX solution. The solution was placed around the portio vaginalis and fornices with a syringe. Women in the control group were irrigated with 20 ml of sterile water.	Women admitted to Lyndon Baines Johnson General Hospital, USA labour and delivery suite at or greater than 36 weeks' gestation	Preterm delivery, foetal distress, malpresentation, intraamniotic infection, cervical dilatation >6 cm and known allergy to CHX.	No significant difference between groups	Not evaluated	No training given, no set protocol e.g. timing	20ml 0.4% CHX	20ml sterile water	CHX group 481 Placebo 466	Maternal outcomes were intraamniotic infection and endometritis. Diagnosis of intraamniotic infection was made if temperature >100°F with two of the following criteria: maternal tachycardia, uterine tenderness, foul-smelling amniotic fluid, maternal leukocytosis, or foetal tachycardia. Diagnosis of endometritis was defined as a postpartum oral temperature >101 ° F, uterine tenderness, and no other source of infection. Patients with a diagnosis of intraamniotic infection could not also be included in the endometritis group.

**Fig. 2:**
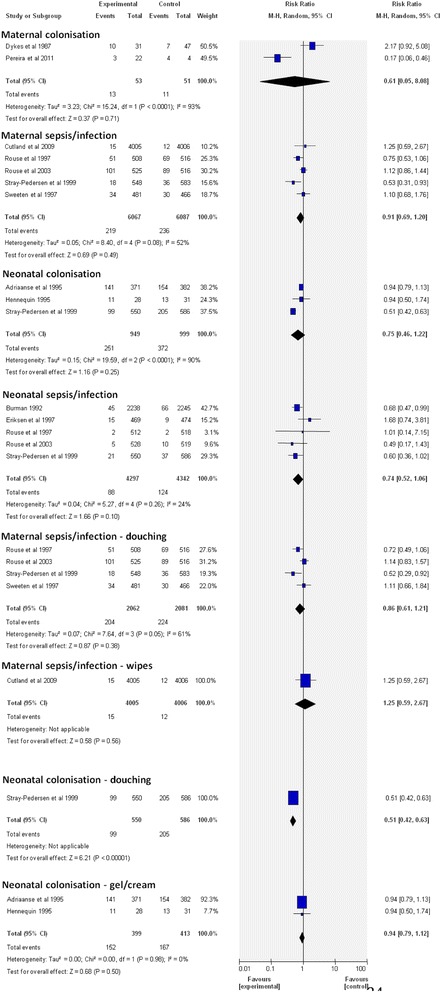
Forest plot comparing the following outcomes and interventions: 1) maternal colonisation; 2) maternal sepsis/infection; 3) neonatal colonisation; 4) neonatal sepsis/infection; 5) maternal sepsis/infection – douching; 6) maternal sepsis/infection – wipes; 7) neonatal colonisation – douching; 8) neonatal colonisation – gel/cream

There was no significant difference in maternal colonization when using vaginal chlorhexidine intrapartum when compared to the control (Fig. 2). Two studies [21, 27] investigated the effect of chlorhexidine on maternal colonization, including 53 participants in the intervention group and 51 in the control group, which also showed no significant difference on colonization (Relative risk (RR) 0.61, 95% confidence intervals (CI) 0.05-8.08) Heterogeneity – I^2^ = 93%, *P* < 0.001.

Five studies [28, 30, 40–42] (Fig. 2) containing a total of 12,154 participants (6067 intervention and 6087 control) did not show a statistically significant effect in maternal morbidity (RR 0.91 95% CI 0.69-1.20) with the chlorhexidine intervention. Heterogeneity – I^2^ = 52%, *P* = 0.08.

The incidence of neonatal colonization was not reduced with any chlorhexidine intervention (Fig. 2). Three studies [22, 42, 43] reported on neonatal colonization on a total of 1948 neonates (949 intervention 999 control) and also showed no reduction in bacterial transmission (RR 0.75 CI 0.46-1.22). Heterogeneity – I^2^ = 90%, *P* < 0.001.

Five studies [20, 29, 30, 41, 42] (Fig. 2) looked at neonatal infection and sepsis. This included 4297 infants in the intervention arm and 4342 in the control group. There was also no reduction with vaginal chlorhexidine (RR 0.74 CI 0.52-1.06). There was significant heterogeneity in the meta-analysis of neonatal colonization (*p* < 0.001, I^2^ = 90%), but no evidence of significant heterogeneity in the meta-analysis of neonatal sepsis/infection as their outcome (*p* < 0.26, I^2^ = 24%). Further analysis of this outcome was undertaken, discriminating between douching and wipes/gel/cream (Fig. 2). The results favoured the douching method, for which the result for neonatal colonization was significant (*p* < 0.001) (Fig. 2). Unfortunately, this particular analysis only contained one study [42].

## Discussion

The meta-analysis did not demonstrate a reduction in maternal colonization or in maternal sepsis/infection when using intrapartum vaginal chlorhexidine cleansing. The incidences of neonatal colonization and neonatal infection/sepsis were also not significantly reduced by this intervention. However, although these results did not show a statistically significant reduction in outcomes, there appeared to be a trend towards a reduction in maternal infection and neonatal colonisation and infection with the douching method, which suggest this subject may warrant further study.

All of the 11 studies reviewed were randomised trials, but seven were assessed to be at high risk of bias in one or more categories. For example, two studies [23, 27] did not perform an intention to treat analysis, which can lead to a failure to preserve randomisation of the groups.

There is significant clinical heterogeneity in the studies analysed (Table 1). In particular, different methods of vaginal cleansing with chlorhexidine were used. In eight studies [20, 21, 27, 30, 41, 42, 44] an irrigation or ‘douching’ method was used, whilst others used gel [23], wipes [40] or cream [22] . In the analysis of these treatment differences, douching was suggested to be more effective, but this may not be a reliable conclusion as only one study [42] with neonatal colonization as an outcome employed irrigation and only one study with maternal sepsis/infection as an outcome [40] used wipes. It is however conceivable that the act of mechanically flushing the vaginal walls could play a part in the physical removal of pathogenic and commensal bacteria. This would oppose the theory that a prolonged contact time found with the use of gel or cream would enhance the bactericidal effects of chlorhexidine.

The use of a control also varied between studies, with three [20, 41, 42] using sterile saline, three [28, 30, 44] using sterile water, one [23] using another placebo and four [21, 22, 27, 40] using no intervention as controls. Aside from the lack of blinding in the non-treatment controls, confounding may have occurred in the use of saline or water. The effect of these controls on vaginal bacteria, whether chemical or mechanical, should be determined.

Some studies included in their analysis the outcomes of mothers who underwent emergency caesarean section [20, 23, 30, 41]. Studies that exclusively focused on women undergoing caesarean section were excluded from our review, but a proportion of women in labour will inevitably require surgical intervention. The intention-to-treat analysis employed may have preserved randomisation, but may also have had an impact on the outcome, as the contamination of the neonate with vaginal bacteria may be less likely if that neonate has not passed through the vagina. Notably, the studies by Rouse et al. [30, 41] also administered one dose of a second-generation cephalosporin to these mothers, which also risks masking the effects of vaginal washing on maternal infection. The same studies also gave prophylactic antibiotics to any mother at risk of early onset GBS infections, which may also have masked both maternal and neonatal complications. In contrast, Burman et al. [20] had ‘GBS carrier status’ as an inclusion criterion (Table 1). In addition, some of the studies did not take account of the duration of labour or prolonged rupture of membranes, which may have led to bias, whilst the Rouse studies [30, 41] administered prophylactic antibiotics to these participants (Table 1).

The studies reviewed also differ in terms of the level of care provider carrying out the intervention, with four [20, 21, 40, 42] using midwives and five [23, 28, 30, 41, 44] using doctors and/or medical students, two unknown [22, 27]. However, the person(s) within each study responsible for performing the intervention (or control, where applicable) varied within the study itself, which may also have influenced outcomes.

The studies reviewed showed heterogeneity for their location. Nine studies were conducted in high-income countries (4 USA, 5 Scandinavia) and only two in developing countries (1 South Africa, 1 Zimbabwe). The Zimbabwean study [27] showed a highly statistically significant result favouring the use of chlorhexidine for the prevention of maternal colonisation. The South African study failed to show a favourable result for the outcome of maternal infection/sepsis. Notably, this study also used vaginal wiping instead of irrigation as the method of intervention, which may be a less effective technique. However, despite such notable heterogeneity between studies, the authors feel that the studies showed sufficient homogeneity in their populations, interventions and outcomes to warrant meta-analysis. It was also felt that the efficacy of the intervention, that is vaginal, intrapartum chlorhexidine, should not be directly affected by the geographical location of the study. Nonetheless, the intervention itself may be economically and technically viable for a low-income setting.

Cochrane reviews [9, 17–19] have previously focused on GBS and other infections separately, concluding that intravaginal/intrapartum chlorhexidine was effective in significantly reducing neonatal colonization with GBS. But they stated that this alone was not sufficient to support the use of the intervention. Our review has also found that, when assessing maternal and neonatal colonization and infectious morbidity of all organisms (excluding HIV) there is no statistical significance to the results, but there is a suggestion that intervention may lead to a reduction in neonatal infection/sepsis.

Goldenberg et al. [38] analysed studies using vaginal chlorhexidine, with or without a neonatal wash, with particular reference to the low income countries. Their analysis of two large, non-randomised studies suggested that one or both of these interventions was successful in improving both maternal and neonatal outcomes. However we believe that it is still useful to separate the two interventions as in our review, to determine the individual effect of each. This is particularly important when considering potential implementation in the low-income countries, where cost-effectiveness and cost-benefit analyses would be of paramount importance, as well as the simplicity of the intervention.

McClure et al. [11] reviewed studies using any chlorhexidine interventions including vaginal, neonatal wipes and umbilical cord cleansing. The group suggested that although several studies reviewed showed promising results, the lack of truly randomized trial evidence stood as a major barrier to implementing the use of chlorhexidine interventions in low-resource settings. Again, we feel that it is advantageous to separate the interventions in order to assess their individual efficacy as exclusive interventions, before combining the outcomes in such a review. Mullany et al. [12] used similar inclusion criteria to McClure et al. [11] for their review, which concluded that although the various chlorhexidine interventions showed promise in reducing neonatal morbidity and mortality, their individual efficacy should be determined before implementation in low-resource settings. We have begun this process in our review, in order to ascertain whether a larger scale randomised controlled trial would be justifiable for the separate intervention of vaginal chlorhexidine washing.

The two Cochrane reviews did this in relation to vaginal, intrapartum chlorhexidine, but may have limited interpretation by separating the causative organisms. As it has been hypothesized that the apparent low prevalence of GBS in low-resource settings may be attributable to under-diagnosis [12], we felt that it was important to conduct our review to include all causative agents.

The Dykes [21], Adriaanse [23], Burman [20] and Stray-Pedersen [42] studies all supported the use of vaginal intrapartum chlorhexidine. All of these studies were conducted in Scandinavian hospitals; therefore the results may not be generalisable to the populations of less developed countries, where a majority of the maternal and neonatal burden of disease exists. Furthermore it is in this setting that the lack of resources and high number of community births make an effective, safe, cheap and low-skill intervention particularly beneficial. In this setting non-randomised studies such as Mushangwe [45] and Taha [46] show promising results.

## Conclusions

Our review shows that intrapartum, vaginal chlorhexidine may lead to a reduction in neonatal infection/sepsis. It is still unclear whether chlorhexidine concentration and method of administration will have a significant impact on outcome, due to the heterogeneity of existing studies. It is therefore our belief that a larger, multicentre, randomised controlled clinical trial in a low-resource setting is justified based on our analysis. Such a trial would require rigorously defined inclusion criteria such as in the Rouse et al. studies [30, 41]. These patients were nulliparous, more than 32 weeks gestation and exclusion criteria were: contraindication to digital cervical examination, active genital herpes, chorioamnionitis prior to randomisation and allergy to chlorhexidine. The studies also carried out double-blinding and computer randomisation.

The use of intrapartum vaginal chlorhexidine should also be considered separately to neonatal skin cleansing, to provide more specific information regarding the efficacy of such interventions. As there are still unanswered question regarding the optimum concentration of chlorhexidine, the frequency and timing (pre/post rupture of membranes) of the intervention and the method used (wipes/gel/cream versus douching), further studies may need to also address these issues.

## Abbreviation

GBS: Group B streptococci
